# Palladium nanoparticle deposition via precipitation: a new method to functionalize macroporous silicon

**DOI:** 10.1088/1468-6996/15/6/065002

**Published:** 2014-11-12

**Authors:** Gilles Scheen, Margherita Bassu, Antoine Douchamps, Chao Zhang, Marc Debliquy, Laurent A Francis

**Affiliations:** 1 SMALL, Institute of Information and Communication Technologies, Electronics and Applied Mathematics, Université catholique de Louvain, Louvain-la-Neuve, Belgium; 2 Max Planck Research Group Micro- and Nanotechnology,Max Planck Institute for Biophysical Chemistry, Göttingen, Germany; 3 Service de Science des Matériaux, Faculté Polytechnique, Université de Mons, Mons, Belgium

**Keywords:** functionalization, macroporous silicon, palladium nanoparticles

## Abstract

We present an original two-step method for the deposition via precipitation of Pd nanoparticles into macroporous silicon. The method consists in immersing a macroporous silicon sample in a PdCl_2_/DMSO solution and then in annealing the sample at a high temperature. The impact of composition and concentration of the solution and annealing time on the nanoparticle characteristics is investigated. This method is compared to electroless plating, which is a standard method for the deposition of Pd nanoparticles. Scanning electron microscopy and computerized image processing are used to evaluate size, shape, surface density and deposition homogeneity of the Pd nanoparticles on the pore walls. Energy-dispersive x-ray spectroscopy (EDX) and x-ray photoelectron spectroscopy (XPS) analyses are used to evaluate the composition of the deposited nanoparticles. In contrast to electroless plating, the proposed method leads to homogeneously distributed Pd nanoparticles along the macropores depth with a surface density that increases proportionally with the PdCl_2_ concentration. Moreover EDX and XPS analysis showed that the nanoparticles are composed of Pd in its metallic state, while nanoparticles deposited by electroless plating are composed of both metallic Pd and PdO_*x*_.

## 1. Introduction

In recent years, hydrogen emerged as the energy vector of the future. Already used in rocket propulsion, hydrogen fueled vehicles (zero carbon emission) are gradually invading the market. Hydrogen is also used in: (i) production of electricity in fuel cells, (ii) storage and transport of energy, (iii) food-processing, and (iv) cooling systems. The growing number of applications is encouraging the development of materials and sensing platforms that allow the rapid and selective detection of this gas. Because of the high solubility of hydrogen in Pd, this metal is a suitable candidate for the design of hydrogen sensing platforms [1].

Pd, and other noble metals like Pt, can be integrated to porous materials for gas sensing. In general, the functionalization of porous materials is bringing them significantly different physical and chemical properties [2, 3]. Also, Pd functionalization will potentially bring an increased selectivity and sensitivity to the targeted gas [4].

For the present study, we are specifically focused on porous silicon that has been chosen among a wide variety of possible substrates [5, 6].

This selection is motivated by the outstanding properties it demonstrates and by the low cost and simple fabrication process via electrochemical etching of silicon. Silicon porosification is a well established process that can be applied in many applications, such as silicon micromachining [7], photonics [8, 9], and sensing [10].

Nonetheless, porous silicon also exhibits semiconducting characteristics leading to its direct application as an electrical transducer in gas sensing [11, 12].

Porous silicon has also attracted considerable attention as a matrix for nanoparticles for both optical and gas sensing applications [13–18].

Porous silicon, like all porous materials, is divided into three categories according to the pore diameter (*d*): nanoporous (*d* < 2 nm), mesoporous (2 nm < *d* < 50 nm) and macroporous (50 nm < *d*). We chose to work with macroporous silicon in order to assure a specific diffusion regime in the pores. Indeed when the mean free path of the gas is comparable to the pore diameter, the diffusion is a combination of Knudsen diffusion and molecular diffusion [19]. At atmospheric pressure, hydrogen not mixed with other gases has a mean free path of the order of 100 nm, which decreases to 60 nm in air. The large diameter of the macropores allows one to have molecular diffusion that is faster than the Knudsen diffusion.

The interactions between catalytic metals and gases are greatly enhanced when the metallic sensing element is nanostructured rather than taken in its bulk form [20]. Thanks to an increased surface-to-volume ratio and higher accommodating surface density of sites, metal nanostructures show an increased catalytic activity. Moreover, nanostructures allow one to suppress phase transitions undergone by certain metals. Those phase transitions can greatly affect the sensing performance of a device over a wide concentration range [20].

A variety of techniques are available for the deposition of palladium nanoparticles: metallic nanofilm deposition followed by thermally-activated dewetting, grafting techniques [20], cluster beam deposition [21], thermal decomposition of salts [22, 23], electrodeposition [24], and deposition via chemical reduction of Pd(II) also commonly called electroless plating [25, 26]. Electroless plating is a cost effective technique based on an auto-catalytic reaction that can be used to deposit films or particles on any kind of substrate. This technique has attracted great interest because it does not require a conductive substrate and any external source of electric current for the deposition to occur. Moreover, electroless plating is simple in operation and requires only a minimal set of equipment. However, despite its simplicity, electroless plating still involves a number of steps and every step needs a careful control of parameters. Moreover, it is a chemical reduction process, and therefore the substrate materials can participate in the reactions giving rise to inhomogeneity of the deposition, especially in compound substrates.

We propose here a deposition via precipitation as an alternative approach to the electroless plating of palladium nanoparticles into macroporous silicon. In contrast to electroless plating, the precipitation process does not involve any redox reaction. Briefly, a porous silicon sample is first immersed in a solution of palladium dichloride (PdCl_2_) and then annealed at high temperature. During the annealing step PdCl_2_ is transformed into Pd. Under a chlorine atmosphere the dissociation temperature of PdCl_2_ is 500 °C while under an inert atmosphere a temperature of 450 °C is sufficient to obtain the precipitation of pure Pd nanostructures [27].

We compare here the two techniques, i.e. electroless plating versus precipitation, in terms of size and shape of the nanoparticles, coating density and homogeneity in the macroporous silicon substrate. Because the morphology of porous silicon and the high roughness of pore walls hinder the application of characterization techniques, we have also performed the same deposition experiments and characterization on standard bulk silicon substrates.

## 2. Materials and methods

### 2.1. Substrates

The samples were prepared starting from 3 inch-diameter p-type silicon wafers (<100>-orientation, 10–25 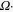 cm, thickness 380 ± 10 *μ*m, Siegert Wafer GmbH). All wafers were cleaned by immersion in Piranha solution (5:2 v/v 98% sulfuric acid: 30% hydrogen peroxide, Chem-Lab) at 110 °C for 10 min followed by a triple rinse in de-ionized water (DI water). The wafers were then immersed in 2% HF (Chem-Lab) solution for 30 s to etch the native oxide followed by another triple rinse with DI water. A 200 nm-thick aluminum layer was evaporated (e-gun, Varian) on the wafer backside. The wafers were then annealed (Lindberg tube furnace) in a H_2_/Ar atmosphere at 432° C to improve the electrical contact between the layer and the Si substrate.

**Figure 1.**
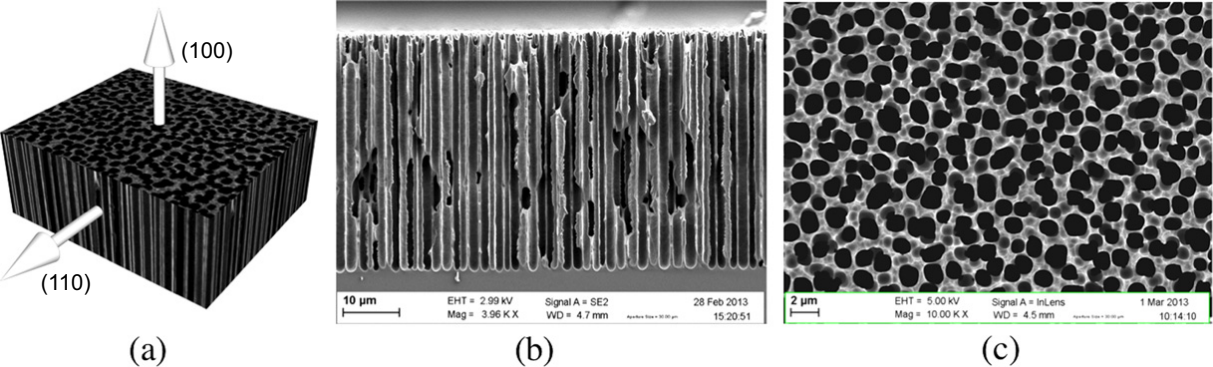
(a) Schematic diagram of a macroporous silicon samples with these crystalline directions representation. (b) SEM cross-sectional view and (c) top view of macroporous silicon layer made by anodization in a HF(48%):DMF = 1:4 solution with a current density of 32.5 mA cm^−2^ for 25 min.

Some of the wafers were anodized in a homemade Teflon single bath cell in order to obtain a macroporous layer. A platinum (Pt) grid was used as the cathode while the anode was the silicon substrate. The electrical contact was made with an aluminum (Al) plate placed in contact with the silicon wafer backside. The previously evaporated Al layer guaranteed the electrical contact between the plate and the wafer. The electrolyte was composed of HF and N, N-dimethylformamide (DMF) (HF(48%):DMF = 1:4, Chem-Lab). The macroporous silicon layer was formed keeping the etching current density *J*_etch_ constant at 32.5 mA cm^−2^ for 25 min (Autolab PGSTAT302N, Metrohm). The macroporous layer so obtained is around 45 *μ*m thick, with a mean pore diameter in between 1 and 2 *μ*m (figure 1). All the wafers, porosified or not, were then diced in 20 × 20 mm rectangular shapes to obtain samples for the deposition experiments. Some of the samples were furthermore etched with a solution of H_3_PO_4_ and isopropanol at 60 °C for 5 min, in order to remove the backside Al layer. After rinsing and drying all the samples were exposed to the ambient air for at least 1 h before the deposition experiments.

### 2.2. Deposition via precipitation

**Figure 2.**
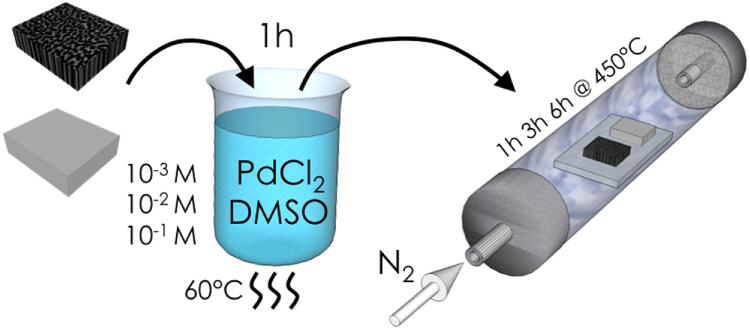
Schematic diagram of the deposition via precipitation experimental procedure.

The precipitation technique consists of two main steps (figure 2):

• immersion of the sample in the deposition solution;
• annealing at high temperature.

The *deposition solution* is composed of palladium dichloride (Pd(II)Cl_2_, 60% Pd, anhydrous, Sigma-Aldrich) dissolved in dimethyl sulfoxide (DMSO, 99.9% A.C.S. reagent, Sigma-Aldrich). DMSO was chosen as solvent instead of water because of its lower surface tension (42.9 mJ m^−2^ compared to 72.7 mJ m^−2^). This allows for better penetration of the solution into the macropores. Three different concentrations for the PdCl_2_ were used: 1 × 10^−3^ M, 1 × 10^−2^ M, and 1 × 10^−1^ M. The solubility at room temperature (RT) of the PdCl_2_ in DMSO is lower than 1 × 10^−1^ M, thus the solution was heated at 60 °C during the experiments. The control of the temperature at 60 °C has two further advantages: the plating, depending on the temperature, is controlled and an higher temperature of the solution helps the diffusion into the pores. The substrates were immersed in the deposition solution for 1 h. Immediately after the immersion, the substrates were annealed at 450 °C in an inert Ar atmosphere (MILA-5000, ULVAC Technologies, Inc.). Three different annealing times were tested: 1, 3, and 6 h.

### 2.3. Electroless plating

Electroless plating is an autocatalytic nucleation and growth process, so a seed is needed to initiate the reaction. Since porous silicon has the ability to chemically reduce many substrates including metal ions as Pd(II) without the need of reducing agents in the solution, a simple method to obtain the nanoparticles on the pore walls, is the immersion of an oxide free porous samples in a solution containing Pd(II) ions. The Pd(II) reduction and the consequent deposition of Pd nanoparticles occur simultaneously with the oxidation of Si to SiO_2_ [25]. However this method leads to a non-uniform deposition and a low surface density of nanoparticles on the pore walls [28].

The uniformity of the deposition is improved by a pretreatment of the porous surface consisting of a sensitization and an activation step [29, 30]. The sensitization of the surface is achieved by immersing the porous samples in a solution containing tin chloride (SnCl_2_). Tin ions show a big affinity for oxide layers and are easily absorbed to form Sn^2+^ clusters. The Sn^2+^ clusters act as seeds for the formation of Pd nuclei, which initiates the deposition of larger structures.

**Figure 3.**
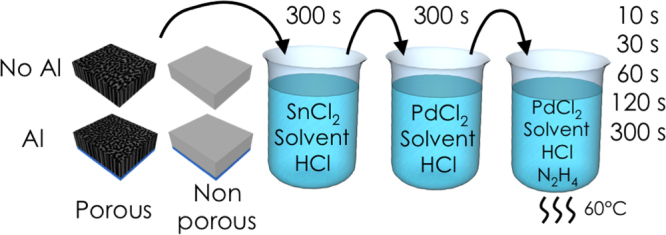
Schematic diagram of the electroless plating experimental procedure.

The technique of electroless plating employed here consists in consecutive immersions in three different solutions (figure 3):

• a sensitization solution;
• an activation solution;
• and a plating solution.

The *sensitization solution* is composed of 3 × 10^−2^ M of tin chloride dihydrate (Sn(II)Cl_2_·2 H_2_O, high-purity reagent, Sigma-Aldrich), 6.77 ml l^−1^ of hydrochloric acid (HCl, 37%, fuming, A.C.S. reagent, Sigma-Aldrich) and a solvent; either DI water or dimethyl sulfoxide (DMSO, 99.9% A.C.S. reagent, Sigma-Aldrich). DI water is the standard solvent used in most electroless deposition processes since it is a very good polar solvent [31]. Also in this case DMSO was chosen instead of DI water as an alternative solvent because of its lower surface tension that allows a better infiltration into the porous layer. HCl is used to regulate the pH solution to 4 since tin(II) only precipitates under acidic conditions. The solution was stirred for 30 min after mixing the reagent. The substrates were immersed in the sensitization solution for 300 s at RT. The purpose of this step is to get Sn^2+^ ions absorbed on the Si walls.

The *activation solution* is composed of 2 × 10^−3^ M of palladium dichloride (Pd(II)Cl_2_, 60% Pd, anhydrous, Sigma-Aldrich), 6 ml l^−1^ of fuming hydrochloric acid (37%) and a solvent; either DI water or DMSO. The presence of HCl ensure a good dissolution of PdCl_2_ in water-based solutions and a better stability of the compound. The solution was stirred for 30 min after mixing the reagent. The substrates were immersed in the activation solution for 300 s at RT immediately after immersion in the sensitization solution. During the activation, the Pd^2+^ ions are reduced on the surface by the Sn^2+^ absorbed ions leading to the formation of Pd^0^ seeds (equation (1))

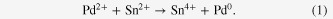
The *plating solution* is composed of PdCl_2_ 2 × 10^−3^ M, 6 ml l^−1^ of fuming hydrochloric acid (37%), hydrazine monohydrate 5 × 10^−2^ M (N_2_H_4_ · H_2_O, 64%-65% N_2_H_4_, reagent grade 98%, Sigma-Aldrich), and a solvent. Two different solvents were used: DI water and DMSO. The solution was heated up to 60 °C. Hydrazine was used as reducing agent for the electroless experiments because this compound has the benefits of being suited to palladium deposition, is active in both acidic and alkaline solutions, and does not leave traces [32]. The solution was stirred for 30 min after mixing the reagents. The substrates were immersed in the plating solution for 10, 30, 60, 120 and 300 s at 60 °C immediately after immersion in the activation solution. All the samples were then quickly rinsed in DI water in order to stop the electroless reaction, and dried with nitrogen.

### 2.4. Characterization techniques

The functionalized samples were characterized by field emission gun scanning electron microscopy (or FEG-SEM, henceforth referred to as SEM), energy-dispersive x-ray spectroscopy (EDX) and x-ray photoelectron spectroscopy (XPS). In porous samples SEM and XPS analyzes were performed on the cross-section obtained by cleaving the sample after the Pd deposition. In non-porosified samples, to better simulate the conditions of a porous substrate, SEM and EDX analyses were performed on the (110) plane face along which the pores are normally grown (figure 1(a)).

A code implemented in Matlab was used to determine the surface density, the dimension and the circularity indices (*C*) of the nanoparticles starting from SEM images. The measurement error is mostly determined by the limited resolution of the SEM images and by the determination of the grey threshold.

## 3. Results and discussion

### 3.1. Deposition via precipitation

#### 3.1.1. Deposition on silicon

**Figure 4.**
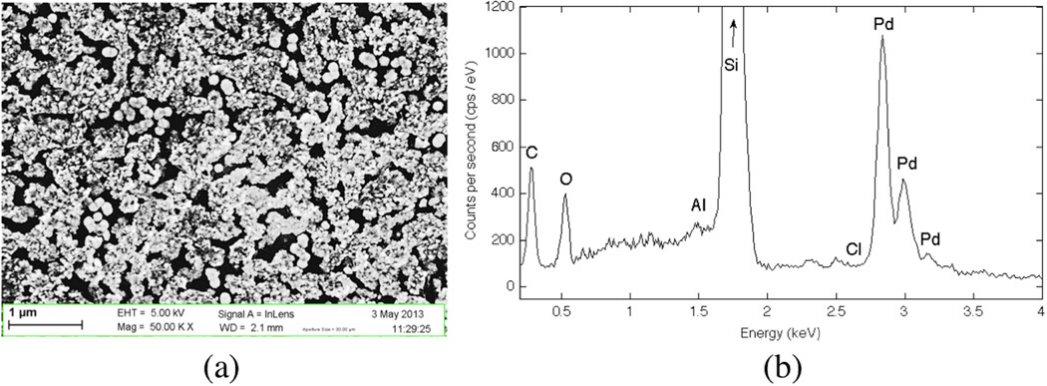
(a) SEM top view and (b) EDX analysis of a silicon sample surface after Pd deposition by precipitation. The following parameters were used for the precipitation: 1 × 10^−1^ PdCl_2_ in DMSO; 1 h immersion; 3 h annealing at 450 °C.

Figure 4(a) shows the SEM view of the cleaved face of a non-porosified silicon sample after the deposition via precipitation in a solution of 10^−1^ M of PdCl_2_. A semi-continuous film composed of interconnected nanostructures entirely covers the surface. The agglomerates exhibit a round-like shape and are superposed on each other. The EDX analysis shows that the elements at higher concentration are respectively Si and Pd (figure 4(b)). The Pd peak is due to the semi-continuous film while the Si peak is due to the substrate. The intensity of the Si peak is bigger than the Pd peaks because the depth of excitation necessary for the EDX analysis is larger than the semi-continuous Pd layer thickness. Peaks for carbon (0.2 keV) and oxygen (0.5 keV) are also noted. The peaks of C and O are attributed to the presence of contaminants on the sample due to the necessary handling. The presence of residual DMSO could also be an explanation. No peak appears around the spectral line of chlorine, suggesting that PdCl_2_ is well-dissociated and gaseous dichlorine was evaporated.

#### 3.1.2. Deposition via precipitation on macroporous silicon

**Figure 5.**
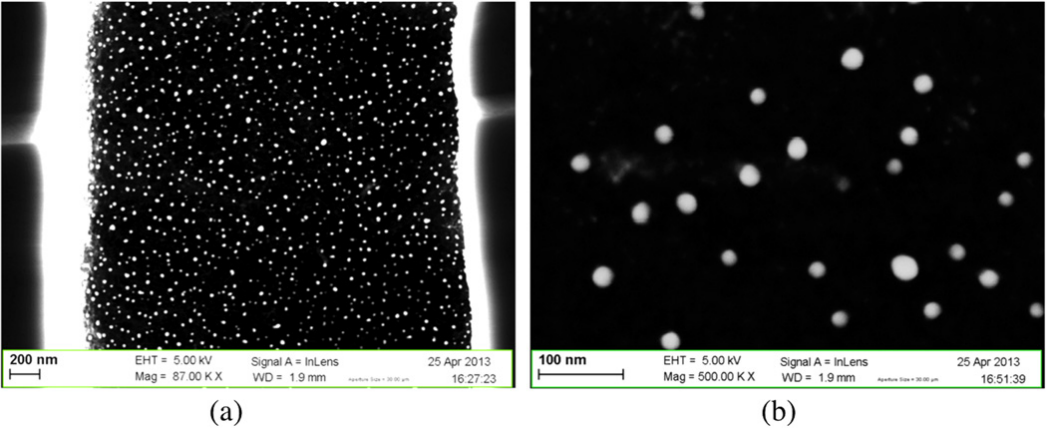
SEM images of Pd NPs deposited via precipitation into the pores: cross-sectional views of part of a pore with nanoparticles deposited on pore walls.

**Figure 6.**
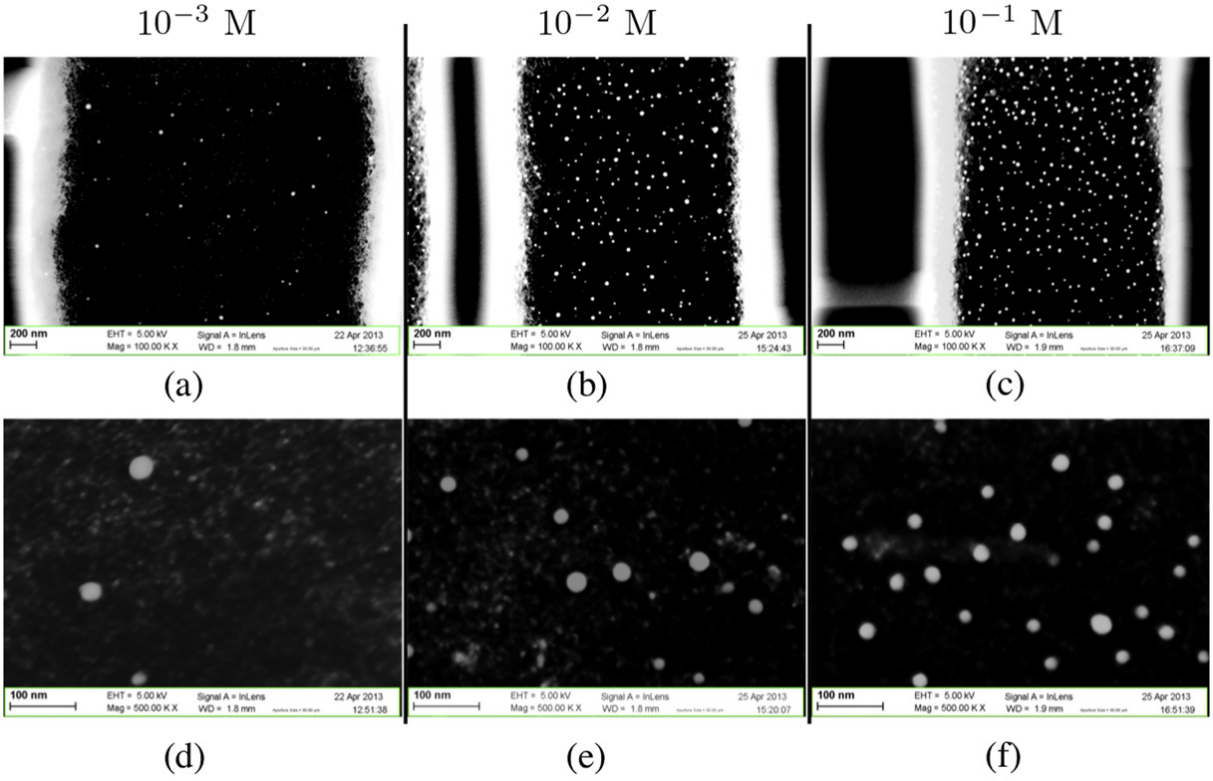
Impact of PdCl_2_ concentration in deposition solution on the deposition via precipitation. Cross sectional views with two different magnifications (up and down) of part of a pore with nanoparticles deposited on pore walls. The samples are immersed in solutions with respective concentrations 10^−3^ M ((a) and (d)), 10^−2^ M ((b) and (e)) and 10^−1^ M ((c) and (f)).

The deposition via this method on macroporous silicon gives rise to a quite different nanostructure morphology. Figure 5 shows two different magnifications of the cross-section of a macroporous silicon sample at the center of a macropore. In this case we obtained the deposition of well-spaced nanoparticles homogeneously distributed in terms of both dimension and surface density. This is most probably due to the smaller amount of reagent that can reach the surface of the pore walls with respect to the open surface of the non-porosified Si samples. The NP diameters range mostly between 15 and 40 nm, rarely exceeding 45 nm. The surface density of nanoparticles is observed as homogeneous on a single pore wall and equally along the entire sample cross-section. It was observed that the surface density of nanoparticles increases with the PdCl_2_ concentration in the deposition solution (figure 6). The samples immersed in the solution with 10^−3^, 10^−2^, and 10^−1^ M exhibit a mean surface density of, respectively, 7, 32 and 118 particles *μ*m^−2^. While the shape of the particles does not change with the PdCl_2_ concentration, a slight change in the size was observed: the average particle diameter increases by increasing the PdCl_2_ concentration and the measured values are 18, 23, and 27 nm.

**Figure 7.**
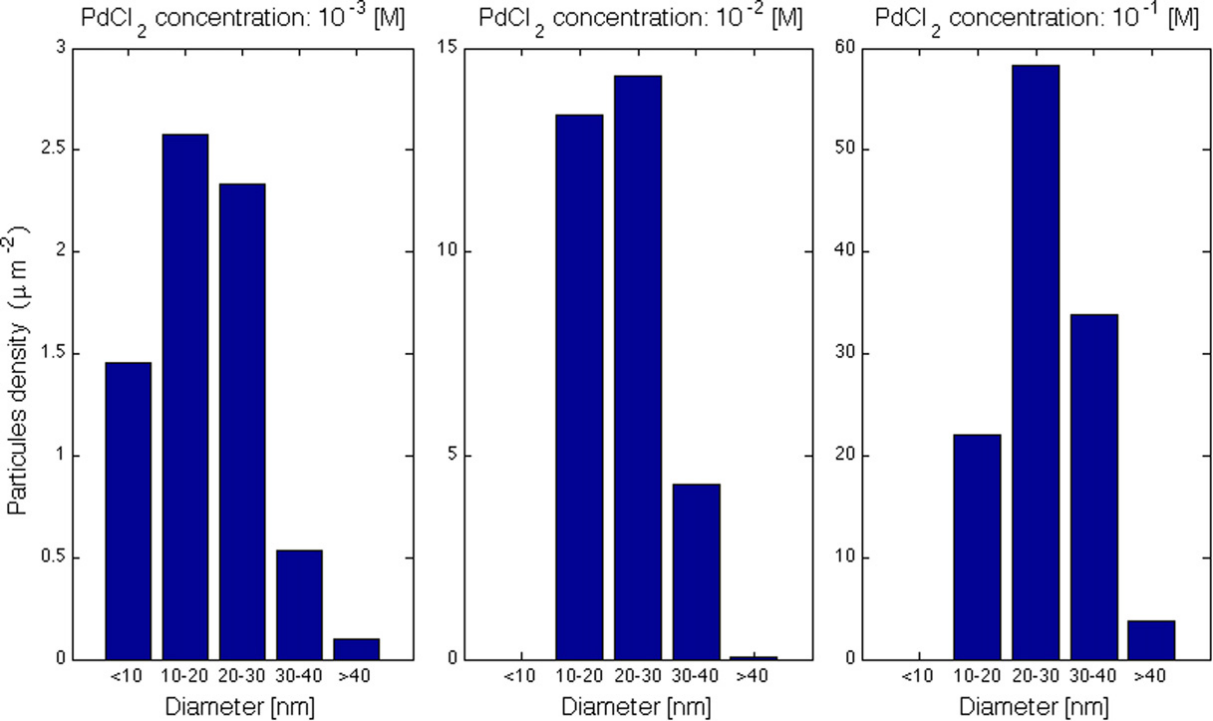
Particle diameter distribution in the form of a histogram of Pd nanoparticles deposited via precipitation.

Figure 7 shows the particle diameter distribution found on the pore walls of three samples immersed respectively in solution containing PdCl_2_ 10^−3^, 10^−2^, and 10^−1^ M. In these histograms it is clearly visible how the PdCl_2_ concentration increase gives rise to the formation of a higher number and bigger size of Pd nanoparticles. Moreover, the concentration increase leads to a reduction of the spread out of the average particle diameter. The maximum value for the nanoparticle diameter remains stable around 45 nm and does not depend on the PdCl_2_ concentration.

Another important parameter to take into account is the duration of the annealing at 450 °C. We analyzed samples after deposition via precipitation in a 10^−2^ M PdCl_2_ solution and 1, 3, and 6 h annealing treatment as the only parameter that has been changed between the experiments. The particle shape was found to be independent on the annealing time, while the computed average particle diameters were 19, 23 and 18 nm respectively for 1, 3, and 6 h of annealing. The computed average particle surface density was found to be respectively 9, 32 and 18 particles *μ*m^−2^. No clear relation between the particle surface density and the annealing time could be highlighted. The homogeneity seems also to be independent on this parameter.

In the deposition via precipitation the formation of nanoparticles does not involve any charge transfer with the substrate. Pd nanoparticles deposited via precipitation are formed in a process of heterogeneous nucleation and growth of spherical PdCl_2_ nanocrystals (colloid crystallization), followed by a thermally induced dissociation of fully-grown PdCl_2_ nanoparticles into Pd nanoparticles. The precipitation starts with the dissociation of the precursor PdCl
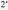
2DMSO. Nanoclusters of PdCl_2_ can form through the agglomeration of sub-nuclei at a nucleation site. Sub-nuclei consist of aggregates of PdCl_2_ of radius smaller than the critical nucleation radius. Nanoclusters smaller than the critical size are very unstable and tend to dissolve. Once a nanocluster reaches the critical dimension, nucleation starts. The reaction continues until the beginning of the actual growing process. The nanoparticle growth is the result of the competition between the addition of sub-nuclei to the nano-structure and the dissolution of the nanoclusters. As a consequence the increase of the PdCl_2_ concentration leads to the formation of bigger and more densely distributed Pd nanoparticles. The growing rate tends to decrease with the particle size, until the process is stopped for a certain critical size of the nanoparticles, which is measured to be around 45 nm for the final Pd nanoparticle.

**Figure 8.**
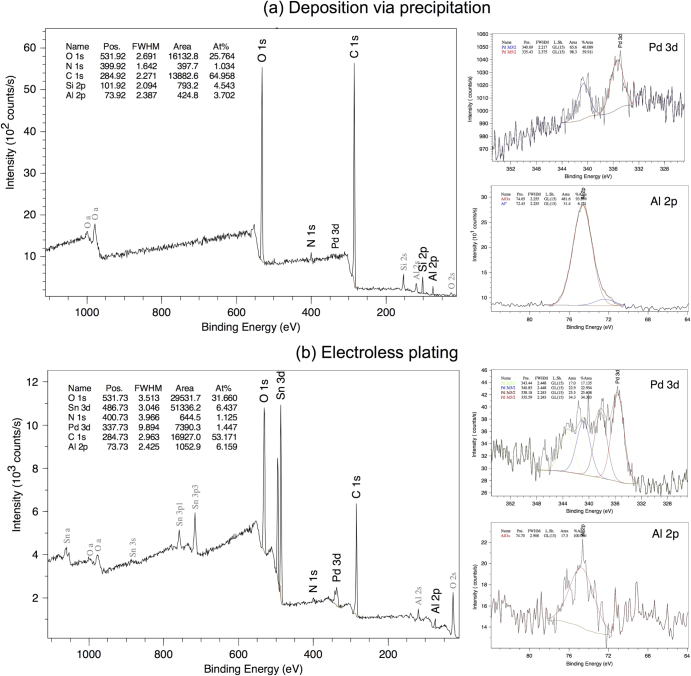
XPS spectra of pore walls after deposition via precipitation (a) and electroless plating (b) of Pd nanoparticles.

The XPS analysis performed on cross-section of porous samples provides more information about the composition of the deposited nanoparticles (figure 8(a)). Overview XPS spectra of the porous films showed signals from oxygen (O), carbon (C), silicon (Si), aluminum (Al), and palladium (Pd). The Al signal originates from the Al layer on the backside of the Si wafer, while the large amount of carbon detected is attributed to the contamination due to the sample handling. The probed area covers the entire cross section of the Si sample. As a consequence Al and contaminants on the sides are also detected. Al is mainly detected in the oxidized state at 74.65 eV, with traces of metallic Al at 72.43 eV. In the region corresponding to the detection of Pd two peaks are clearly visible at 335.43 and 340.69 eV, both attributed to Pd in its Pd^0^ state. In all samples no traces of chlorine were detected, which, together with the results of the EDX analysis performed on non-porous samples, proves that PdCl_2_ is completely dissociated during the thermal treatment. No difference between samples annealed for 1, 3 and 6 h was observed in the XPS analysis.

### 3.2. Electroless plating

In contrast to the deposition via precipitation, electroless plating is not only affected by experiment-related parameters, but also by the properties of the substrate. As we observed that the Al backside layer plays an important role and strongly affects the deposition results, we conducted Pd electroless plating on samples with and without Al layer on the backside.

#### 3.2.1. Pd electroless plating on bulk silicon

**Figure 9.**
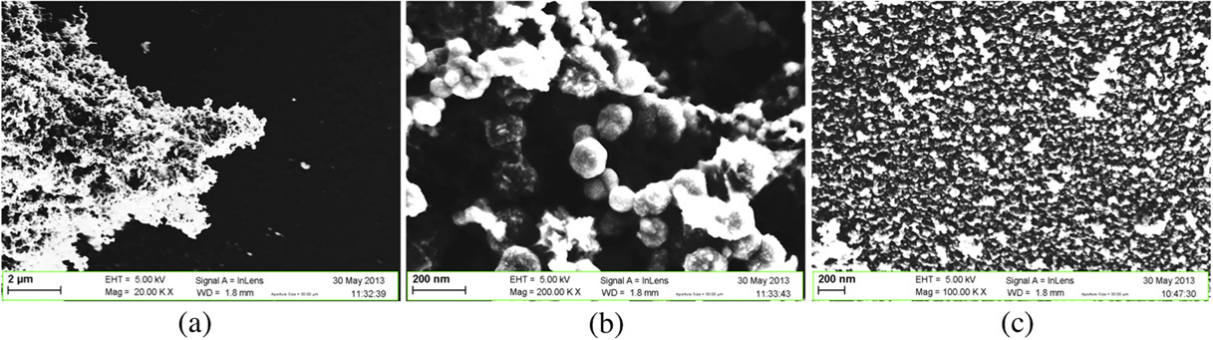
SEM images of deposits formed by electroless plating on flat silicon surface ((a) and (b)) without and (c) with aluminum on the wafer backside face. In the absence of aluminum on the backside face ((a) and (b)), the deposit is not a continuous layer. In the presence of aluminum in the backside face (c), the deposit is a compact and homogeneous film which covers the entire surface.

Figures 9(a) and (b) show two different magnifications of the cross-section of a non-porosified Si sample after Pd electroless plating. No Al layer was on the backside of the analyzed sample. The result of the electroless plating is a non-continuous layer that leaves large portions of the silicon surface uncovered. The higher magnification SEM image (figure 9(b)) shows that the layer is actually composed of round-shaped agglomerates grown one close to another. Figure 9(c) shows an SEM view of the cross-section of a silicon sample with Al on the backside. As for samples without Al we obtained a layer composed of Pd round-shaped nanostructures agglomerates, but the layer is more homogeneous and covers the entire silicon surface.

**Figure 10.**
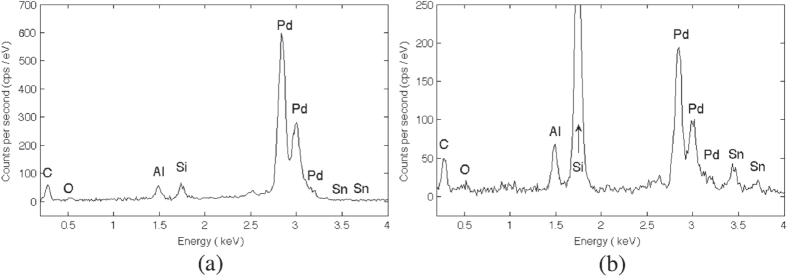
EDX analysis of a silicon sample surface after Pd deposition by electroless plating (a) without and (b) with aluminum on the wafer backside face.

We performed EDX analysis of both samples. Figure 10 shows the EDX spectra of a bare silicon sample (a) and of a sample with Al on the backside (b). In both spectra, the peaks corresponding to carbon (0.277 keV), oxygen (0.525 keV), aluminum (1.486 keV), silicon (1.739 and 1.836 keV) and palladium (2.839 and 3.172 keV) are visible. The presence of O and C can be attributed to contamination due to the handling of the samples. The very low intensity of the Si peak in samples of bare silicon leads to the assumption that in these samples the Pd layer is thicker than the penetration length of the beam. The same assumption could explain why no trace of Sn was found on bare silicon samples while in samples with Al on the backside two small peaks close to the Sn spectral lines (3.443 and 3.662 keV) are visible in the spectra. The thickness of the Pd layer on samples with Al on the backside is probably smaller than the penetration depth of the beam into the silicon substrate. Residual Sn was expected to be found when the beam goes through the Pd layer, which is not seen.

#### 3.2.2. Pd electroless plating on macroporous silicon

**Figure 11.**
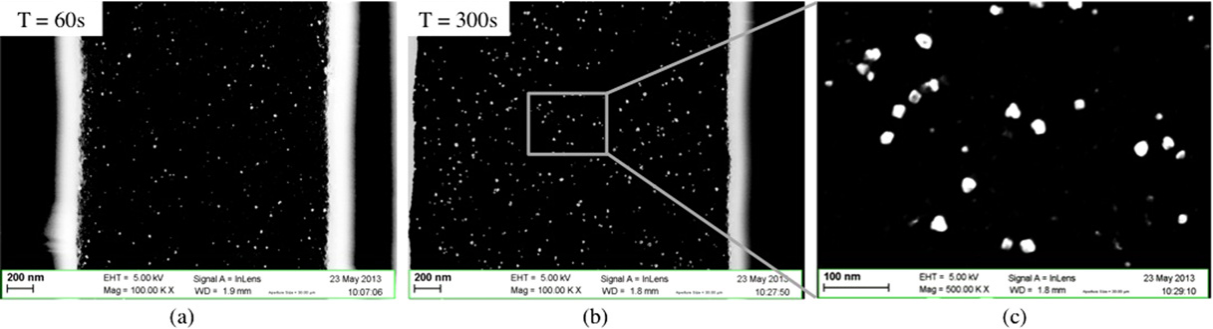
SEM images of cross sectional views of part of a pore with nanoparticles deposited on pore walls by electroless plating (sample without aluminum on backside face).

Figure 11 shows two cross-sectional views of a macroporous sample (magnification of a single pore wall) on which the Al layer was removed before the Pd electroless plating. The two samples were immersed in a plating solution with DI water as solvent for 60 s (figure 11(a)) and 300 s (figures 11(b) and (c)).

Although electroless plating resulted in the deposition of Pd nanoparticles, in contrast with the deposition via precipitation technique, the nanoparticles are distributed very unevenly on the pore walls. In certain areas of the pores, no particles could be found. As for the section of the pores exhibiting particles, the surface density is around 15 particles *μ*m^−2^ for the samples immersed in the plating solution for 60 s, and 37 particles *μ*m^−2^ for samples immersed for 300 s. Aggregates of 2, 3 nanoparticles were also found. Contrary to our expectations, the computed average diameter is of around 19 nm for both samples. The time in the plating solution should in fact determine the size of the nanoparticles: the reaction of Pd deposition is supposed to continue until the reactants are present in the solution, on the sites that have been activated in the previous step (the sensitization and activation times were 300 s each for both samples).

In both samples the particle shape is variable. A number of round-shaped particles are observed, but most of them have irregular shapes, and others are the clear result of the overlap of different particles (figure 11(c)). The NPs surface density exhibits large variation along the pore depth. The same kind of inhomogeneity was observed between pores. In some of the pores no NPs were deposited.

**Figure 12.**
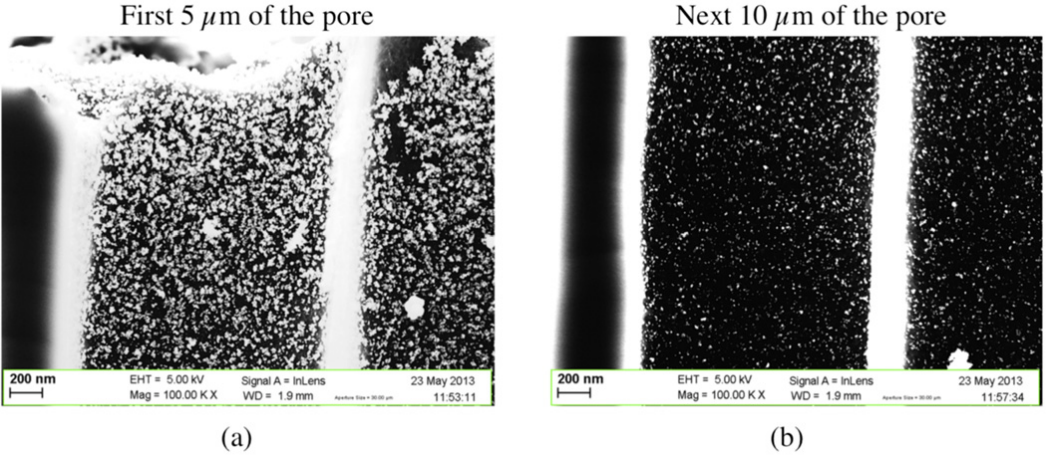
SEM images of cross sectional views of part of a pore with nanoparticles deposited on pore walls by electroless plating (sample with aluminum on backside face). Views of cross-section at pore opening (a) and at 15 *μ*m in depth (b).

The presence of Al on the sample backside significantly affects the results of the electroless plating into the macropores. Figure 12 shows the cross section of a macroporous silicon sample in which the Al layer was still on the backside of the sample. Two portions of a macropore are shown, at the entrance (figure 12(a)) and at 15 *μ*m in depth (figure 12(b)). In samples with Al on the backside a high surface density of nanoparticles is found at the entrance of the pore, in the first 5 *μ*m in depth. Aggregates of particles that almost form a continuous layer are found in this area of the pores. The overall surface density (7 particles *μ*m^−2^) is on the other hand comparable and even smaller than that computed for samples without Al. In fact, the surface density of particles decreases considerably with the pore depth and almost no particles can be found after 15 *μ*m in depth. The mean particle diameter was found to be 29 nm, with small variation around this value. Also in this case particles with different shapes can be found. Round-shaped particles can be rarely found. Tests carried out using DMSO instead of DI water did not give rise to significantly different results, just a slightly better uniformity in the NP distribution was observed.

The presence of the Al layer on the sample backside is the parameter that most affects the results of the electroless plating deposition experiments. We can say that we are in the presence of Al-assisted Pd electroless deposition. The oxidation of the Al film proves that electrons are injected into the silicon substrate. These electrons reach the surface, and are then used for the reduction of Sn and Pd nuclei at the surface during the sensitization and activation steps. In other words, Al acts as a reducing agent promoting the deposition of active nuclei during the first two stages of the electroless deposition reaction. The Al layer was dissolved without formation of hydrogen bubbles while the sample was immersed in the deposition baths. No gaseous hydrogen can be formed because the required electrons are instead injected in the bulk silicon. Moreover the higher surface density of the deposited film in bulk silicon samples, and at the entrance of macroporous layers in samples with Al on the backside, could be explained by a change occurring in the first two steps of the deposition, because the Pd plating occurs only on pre-deposited and activated sites.

The results of the XPS measurements give more information about the composition of the deposited nanoparticles. Figure 8(b) shows the spectra of a sample in which was present an Al layer on the backside that was etched during the electroless plating. The XPS spectrum shows the presence of O, C, Si, and of more interest Al, Sn and Pd. Although the Al layer on the backside was completely etched during the electroless plating, traces of Al are still present on the samples, it has to be noted that only the oxidized state of Al was found. Most probably, the Al^3+^ ions resulting from the dissolution of the Al layer in the electroless plating process, react and bond with the SiO_2_ layer on the surface. The presence of a large amount of Sn suggests that Pd does not substitute most of the Sn clusters. Assuming that the Sn cluster size is limited, this large amount of Sn also suggests that the macropores walls are fully or partially covered with Sn clusters in the sensitization step. In next steps, these Sn clusters are not used for the following formation of Pd nanoparticles, which is limited to the entrance of the pores.

The decomposition of the XPS spectrum at binding energies corresponding to Pd shows the presence of two peaks at 335.59 and 340.85 eV corresponding to Pd in its metallic state and other two peaks at 338.18 eV and 343.44 eV attributed to Pd in its oxidized state (PdO_*x*_ or PdCl_*x*_). The low amount of Cl detected, less than the 0.1% suggests that PdO_*x*_ dominates.

Table 1 summarizes the results obtained, for macroporous silicon samples processed with deposition via precipitation and electroless plating, using the image processing assisted analysis. In the deposition via precipitation, the PdCl_2_ concentration plays an important role and affects many of the characteristics of the deposited nanoparticles: (i) the surface density of particles increases with the PdCl_2_ concentration, (ii) the mean particle diameter increases with the PdCl_2_ concentration, also the number of particles larger than the average size increases proportionally to the concentration, (iii) the maximum in diameter does not depend on the concentration and is stable around 45 nm, and (iv) in the range of concentration for PdCl_2_ that we used, the deposition via precipitation allows to homogeneously cover the entire pore walls with Pd nanoparticles.

**Table 1.** Summary of the characterization of Pd nanoparticles deposited into the porous layer by deposition via precipitation and electroless plating. The shape quality of the deposited nanoparticles are described by the symbols ‘+’ for spherical and ‘−’ for non spherical. The characterization of the electroless plating with Al was made by excluding the first 5 *μ*m, which are coated by interconnected nanoparticles.

	Avg. NP diameter (nm)	NP shape (+/−)	Avg. NP surface density (part.*μ*m^−2^)	Surface density standard deviation (part.*μ*m^−2^)
**Dep. via prec.**				
10^−3^ M; 3 h	18	+	7	8
10^−2^ M; 3 h	23	+	32	4
10^−1^ M; 3 h	27	+	118	47
10^−2^ M; 1 h	19	+	9	5
10^−2^ M; 6 h	18	+	18	7
**Elec. plating**				
without Al	20	−	25	23
with Al (excluding	29	−	7	88
the first 5 *μ*m)				

The results obtained by electroless plating are very different. Although the NP average size is very similar to that obtained with the deposition via precipitation, their shapes are mostly not spherical and a large range of different shapes can be found. The nanoparticle surface density on the pore walls is very low and variable over the pore depth and in-between pores.

## 4. Conclusions and perspectives

In conclusion, the deposition via precipitation proposed here was found to be an effective method to functionalize macroporous silicon with Pd nanoparticles. Round-shaped and uniformly distributed nanoparticles were deposited on the surface of the pore walls. Tunability of the number of nanoparticles on the surface can be achieved by changing the PdCl_2_ concentration in the deposition solution. The EDX and XPS analysis showed the presence of metallic Pd in the pores, and no chlorine was detected. The results are particularly encouraging in comparison to those obtained with one of the most standard techniques for the deposition of Pd nanoparticles, electroless plating. It was in fact shown that, with this technique, it is not possible to obtain a uniform coating of the pore walls with Pd nanoparticles. Moreover EDX and XPS analysis showed the presence of Sn and Al contamination on the deposited layer, and the presence of Pd in its oxidized state.

The deposition via precipitation not involving any redox reaction is compatible with a large variety of materials and could thus be used to functionalize different porous materials and sensing platforms. Future work will be devoted to developing a theoretical model that describes the mechanisms involved in the nucleation and growth of PdCl_2_ nanoparticles, lately dissociated into Pd nanoparticles by thermal treatment. Moreover the deposition via precipitation will be applied to functionalizing a macroporous silicon based sensing platform in order to investigate the otherwise unpredictable effectiveness of the method in improving sensitivity and selectivity to hydrogen.
